# RESEARCH INITIATIVES: What Lies Ahead for Nanotechnology?

**DOI:** 10.1289/ehp.117-a146

**Published:** 2009-04

**Authors:** Graeme Stemp-Morlock

Nanotechnology, the engineering of functional materials at an atomic or molecular scale, has been among the fastest growing fields of science and technology. Worldwide sales of products incorporating nanotechnology are projected to total $2.6 trillion by 2014, according to a 2004 report from Lux Research titled *Sizing Nanotechnology’s Value Chain*. But with the increasing use of nanomaterials in many consumer products has come growing concern about potential environmental, health, occupational, and general safety hazards. At the 2009 annual meeting of the American Association for the Advancement of Science, scientists held a seminar titled “Driving Beyond Our Nano‐Headlights?” to discuss recent nanotoxicologic research as well as health and environmental policy implications of the use of nanomaterials.

Speaker Agnes Kane, a medical professor at Brown University, revisited the analogy between chrysotile asbestos fibers and carbon nanotubes (CNTs), a concept first introduced in 1998. In collaboration with fellow Brown University researcher Robert Hurt, Kane has compared the two materials and found similarities in surface area, physical properties, and geometry, raising the possibility that CNTs may show asbestos‐like behavior in the human body. Also, using a newly designed cell culture model, Vanesa Sanchez, a graduate student in Kane’s laboratory, found that very low doses of CNTs (1 μg/mL) appeared to cause lesions known as granulomas similar to what occurs with asbestos fibers. Moreover, the CNTs formed a cage‐like structure that Kane suspects might promote granuloma formation.

Kane also cited research by Ken Donaldson and colleagues of the University of Edinburgh in which the mesothelial lining of the mouse body cavity was exposed to CNTs. (In humans, asbestos is known to cause mesothelioma, a rare form of cancer of this lining.) As Donaldson’s group reported in the July 2008 issue of *Nature Nanotechnology*, this exposure resulted in asbestos‐like pathogenic effects that included inflammation and granuloma formation.

Sanchez’s new cell culture model is one of the few that can screen for potential adverse health effects of nanomaterials. Along these lines, the various challenges of conducting nanotoxicologic research are a central focus for Sally Tinkle, senior science advisor in the Office of the NIEHS Director. Tinkle said the shape and manufacture of a nanoparticle will have a profound impact on the particle’s reactivity, as well as how it interacts in the body. “Nanomaterials hold incredible promise to solve significant world problems, like the need for energy and clean water, but these new materials have novel physical and chemical properties, and we don’t know yet what their interactions with biological systems will be,” she said.

Tinkle noted various problems with the interpretation of toxicologic data for nanomaterials. Citing correspondence published in the February 2007 issue of *EHP* by Günter Oberdörster and colleagues at the University of Roch‐ester, she pointed out that toxicologic data could be interpreted differently depending on whether you looked at the nanomaterial’s mass or surface area. Moreover, in six common toxicology assays on the same nano‐material sample conducted by Nancy Monteiro‐Riviere and colleagues at North Carolina State University, Tinkle noted that some assays showed a significant effect of exposure whereas others showed none. CNTs were also found to compromise the accuracy of the assays, but nanotoxicologists have yet to identify adequate positive and negative controls that would better reveal the presence and effects of such interference.

Norris Alderson, associate commissioner for science at the U.S. Food and Drug Administration (FDA), raised a related issue in the manufacturing domain—that of guaranteeing consistency (primarily in terms of size, shape, and purity of composition) from batch to batch of nanomaterial to ensure safety and efficacy. “Let’s say you’ve got a nanoscale material for which the majority of particles is 50 nm,” said Alderson. “But if there’s variation on both sides of that, how much can you vary that distribution and still procure a material with the same characteristics or safety and efficacy?”

Establishing appropriate standards for nanomaterial production is a salient concern for Travis Earles, National Science and Technology Council representative on the nanotechnology portfolio in the White House Office of Science and Technology Policy. Earles stated that such standards were still a work in progress, both nationally and internationally. “It’s a little bit of the Wild West in the standards development side of things,” he said. “The standardization effort is quite crucial because it ultimately will be the context through which we can successfully or unsuccessfully innovate nanotechnology into commercial use.”

Despite these and other challenges, Earles was able to sound a note of optimism for scientists who are looking toward whatever might lie beyond the nano‐headlights: Federal funding allocated specifically for environmental, health, and safety research in nanotechnology has grown from $34.8 million in 2005 to $58.6 million in 2008. The multiagency National Nanotechnology Initiative anticipates that funding for the current year will increase to $76.4 million, well above the amount of direct investment made by any other country.

## Figures and Tables

**Figure:**
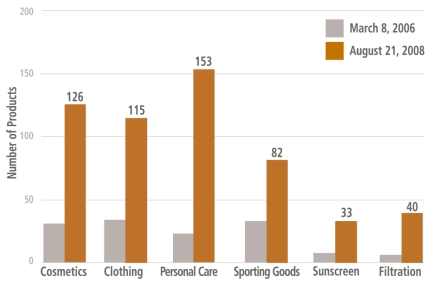
Health and fitness products lead the way in terms of nanotechnology market growth. Between 8 March 2006 and 21 August 2008, the number of “nano-enabled” health and fitness products on the market approximately quadrupled. Source: The Project on Emerging Nanotechnologies, http://www.nanotechproject.org/inventories/consumer/

